# Associations between maths ability mindset, maths task preference and physiological responses to feedback during an arithmetic task across two primary school years

**DOI:** 10.1111/bjep.70115

**Published:** 2026-08-07

**Authors:** Ita Puusepp, Tuisku Tammi, Tanja Linnavalli, Minna Huotilainen, Sonja Laine, Kirsi Tirri

**Affiliations:** ^1^ Faculty of Educational Sciences University of Helsinki Helsinki Finland; ^2^ Cognitive Science, Department of Digital Humanities University of Helsinki Helsinki Finland; ^3^ Cognitive Brain Research Unit, Faculty of Medicine University of Helsinki Helsinki Finland; ^4^ Centre of Excellence in Music, Mind, Body and Brain, Faculty of Educational Sciences University of Helsinki Helsinki Finland; ^5^ Present address: Faculty of Behavioural and Movement Sciences Vrije Universiteit Amsterdam Amsterdam The Netherlands

**Keywords:** feedback, habituation, longitudinal, maths, mindset, primary school, skin conductance

## Abstract

**Background:**

Students' mindsets, reactions to feedback and learning‐related behaviour are important for academic success, yet little is known about how these factors and their interconnections develop during primary school.

**Aims:**

To explore longitudinal associations between students' mindsets, physiological responses to performance feedback, and task preference in maths across two primary school years.

**Sample:**

Eighty‐nine students were followed from grade 3 to grade 4.

**Methods:**

During the autumn term of both grades, students' mindsets were assessed using a questionnaire. After a challenging maths task, students chose their next task—either another challenging one to learn more or an easy task to succeed. Skin conductance was recorded during an arithmetic task with performance feedback. Mixed‐effects models were used to analyse associations and their development.

**Results:**

We found that growth‐minded students preferred the challenging task in both grades, yet mindset did not relate to feedback‐related physiological responses. Task preference demonstrated only partly marginal associations with these responses and only in the beginning of the task. Specifically, students preferring the challenging task showed a smaller and faster‐habituating physiological reaction to feedback at task onset.

**Conclusions:**

Physiological responses and mindset had unique relations with task preference, suggesting the potential importance of both in learning. Associations with only initial physiological responses and their habituation, not overall feedback‐related responses, suggest subtle temporal changes in physiological reactions during educational activities. The present findings, although requiring replication, suggest that supporting students in regulating performance‐related arousal at task onset may be particularly important.

## INTRODUCTION

Encountering feedback and making learning choices form part of students' daily school‐related experiences. Importantly, both have a profound effect on their learning and future attainment (Andersson & Bergman, 2011; Wisniewski et al., 2020). However, the effect of feedback is far from homogeneous (Wisniewski et al., 2020), and student characteristics have been highlighted as a central element of the feedback process (Panadero & Lipnevich, 2022). Researchers examining the factors influencing students' reactions to feedback and their learning choices have identified mindsets—beliefs about the malleability of abilities—as a highly important factor shaping both (Janssen et al., 2022; Puusepp et al., 2023; Yeager & Dweck, 2012). Existing research suggests that primary school may be an especially important period concerning the development of students' mindsets and the interrelations between these beliefs, reactions to feedback and learning‐related behaviour (Kinlaw & Kurtz‐Costes, 2003, 2007; Puusepp et al., 2023). However, to our best knowledge, no studies have longitudinally examined the interrelations between all three factors during primary school. A better understanding of these associations and their development could inform educators on how to better support the development of beliefs and behaviours among students that promote learning. Furthermore, a better understanding of students' largely involuntary physiological reactions to feedback and how these relate to their beliefs and behaviour could inform educators on when to anticipate and manage students' excessive arousal, creating a more supportive learning environment.

Therefore, the present study explores the associations between primary school students' self‐reported mindsets, physiological responses to feedback and behaviourally assessed task preference across two primary school years. We examine these links and their development in the context of mathematics. Maths is a subject where success is considered, more than some other domains, to depend on innate ability (Heyder et al., 2020; Leslie et al., 2015). Furthermore, students consider maths to be one of the most important school subjects (Dundar & Rapoport, 2014).

### Mindsets in learning

Beliefs about the malleability of human abilities (e.g., intelligence) are referred to as mindsets (Dweck, 2000). Mindsets as motivational dispositions range from fixed to growth, with the former referring to the belief that human abilities are static and innate and the latter denoting the belief that abilities are malleable and can be developed. Students with a fixed mindset tend to interpret failure and the need to exert effort as indicators of a lack of innate ability (Blackwell et al., 2007; Hong et al., 1999; Tempelaar et al., 2015). This leads fixed‐minded students to avoid situations that threaten their high‐ability image and to react maladaptively when faced with setbacks (Yeager & Dweck, 2020). Conversely, students with a growth mindset view setbacks as informative about what remains to be learned and effort exertion as an inherent part of learning (Blackwell et al., 2007; Yeager & Dweck, 2012). Thus, growth‐minded students are more likely to seek challenges and invest more effort in learning (Janssen et al., 2022).

The detrimental effects of a fixed mindset are suggested to emerge primarily when students face a competence threat—when they risk losing their reputation for high‐ability (Yeager & Dweck, 2020). Supporting this, the meta‐analysis by Burnette et al. (2013) indicates that while a growth mindset is generally related to mastery‐oriented strategies, these benefits become significantly more pronounced when competence threat is involved. Because negative feedback on one's performance inherently carries information threatening one's perceived competence, researchers have focused on the links between mindset and reactions to such feedback. Specifically, research indicates that holding a fixed mindset leads students to perceive such feedback as threatening (Zingoni & Byron, 2017), plan to disengage and avoid skill‐improving activities (Hong et al., 1999; Hu et al., 2017), while prioritizing restoring one's self‐esteem instead (Nussbaum & Dweck, 2008).

While the associations described above are well‐documented, research among young students suggests that some of such theoretically assumed associations may only develop during primary school years. As such, research has shown that while 4th grade students' preference for easy and challenging tasks is linked to their mindsets, this is not the case among younger students (Kinlaw & Kurtz‐Costes, 2007). Similarly, Puusepp et al. (2023) demonstrated that by grade 4 students' mindsets are related to their attentional processing of feedback, while in grade 3 no such associations were observed. These results suggest that, during primary school years, young students' mindsets, their reactions, and behaviours may be developing towards greater coherence. In line with this speculation, primary school has been shown to be an important period where beliefs about intelligence undergo notable changes (Kinlaw & Kurtz‐Costes, 2003). Nevertheless, the findings concerning the development of these associations during primary school years are tentative and require replication.

### Feedback‐related physiological arousal

The sympathetic nervous system is responsible for preparing the body for action. It is activated upon the perception of motivationally significant or emotionally arousing events (Kreibig, 2010), with such activation being largely involuntary. This activation is reflected in numerous physiological indicators and one of the most widely studied sensitive indicators of the activity of the sympathetic nervous system is electrodermal activity (EDA)—changes in the electrical properties (conductance/resistance) of the skin that result from sweat secretion by eccrine sweat glands (Dawson et al., 2007). EDA is often divided into a slower fluctuating tonic component—skin conductance level, which reflects general states of arousal and alertness—and a rapidly changing phasic component—skin conductance response (SCR). SCRs encompass rapid changes in skin conductance triggered by specific events. Because they reflect reactions to brief, discrete stimuli, they are well‐suited to studying responses to specific stimuli during a task (Boucsein, 2012; Dawson et al., 2007). SCRs are elicited by motivationally significant stimuli (e.g., Barry, 2004; Dindo & Fowles, 2008; Zimmer & Richter, 2023). This motivational significance can result from task‐ or self‐relevance of the stimulus, but also from properties such as novelty, unexpectedness or intensity. Greater motivational significance of a stimulus, encompassing its novelty or task‐relevance, is related to the greater magnitude of the SCR (Bradley, 2009), with this magnitude also related to the subjective arousal ratings of the stimuli (e.g., Wang et al., 2018). Moreover, such responses are also elicited by anticipation of personally significant stimuli, such as outcomes or feedback (see Dawson et al., 2011).

The motivational significance of a stimulus affects not only the magnitude of the SCR but also the rate of the habituation of this response—the progressive decrease of this response during repeated presentations of the stimulus (Rankin et al., 2009). Specifically, the SCR habituates slower when the repeatedly presented stimulus is arousing or task‐related than when it is neutral or unrelated to the task (Barry, 2004; Öhman et al., 1974; see also Bradley, 2009).

### Mindsets and feedback‐related physiological responses

As described above, the associations between mindsets and reactions in situations involving setbacks or a competence threat are rather well‐documented by studies relying on self‐reported and behavioural data. In contrast, psychophysiological research into these reactions is still surprisingly scarce. The few existing studies, however, are in line with the findings based on self‐reported and behavioural data. For example, in their fMRI study, Bejjani et al. (2019) showed that, under competence threat, a fixed mindset correlates with neural patterns reflecting a heightened punishment response to negative feedback. Similarly, Mangels et al. (2006) found that a fixed mindset predicts larger brain responses to such feedback, suggesting increased attention towards evaluative feedback. While these studies focused on adults, Puusepp et al. (2023) examined primary school students, with longitudinal data suggesting that the associations between mindset and neural responses to feedback emerge during primary school. Specifically, while mindsets were not related to brain responses in third graders, a fixed mindset predicted enhanced attentional processing of both positive and negative feedback in fourth grade (Puusepp et al., 2023). Nevertheless, the findings on the links between mindsets and psychophysiological responses, especially those based on studies conducted among primary school students, are tentative and require replication.

In this study, we focus on SCRs in relation to feedback. According to mindset theory, students with a greater fixed mindset are more concerned about their performance as they are inclined to view their performance as indicative of innate ability or its absence. Therefore, mindset as a motivational disposition could be expected to shape physiological arousal during performance feedback. We focused on feedback‐related SCRs during an arithmetic task. Performance‐related feedback could hold greater motivational significance for fixed‐minded students, and this could be reflected in greater feedback‐related SCRs compared to other task‐relevant stimuli (e.g., the appearance of the task) on average as well as the slower habituation of these feedback‐related SCRs. Furthermore, if greater feedback‐related SCRs reflect greater activation of the defensive motivational system (e.g., fear of failure), these responses could also be expected to be related to avoidance of challenging tasks—a behaviour often linked to fixed mindset, especially in situations where students risk losing their reputation for high‐ability (Yeager & Dweck, 2020). More specifically, such increased arousal can lead students to prefer easy tasks where success is guaranteed over challenging tasks entailing the risk for further failure in order to reduce their (negatively valenced) arousal.

### Present study

Building on the research that has demonstrated links between mindsets, effort beliefs, interpretation of setbacks and feedback, and learning behaviour in the presence of competence threat, this study examined how feedback‐related physiological arousal relates to mindsets and learning behaviour. More specifically, the present study aimed to explore associations between primary school students' self‐reported maths ability mindsets, their behaviourally assessed maths task preference, and physiological responses to performance feedback in maths over two consecutive school years. Figure 1 summarizes the theoretical differences between a growth and fixed mindset and shows how we operationalized the focal constructs in this study.

**FIGURE 1:**
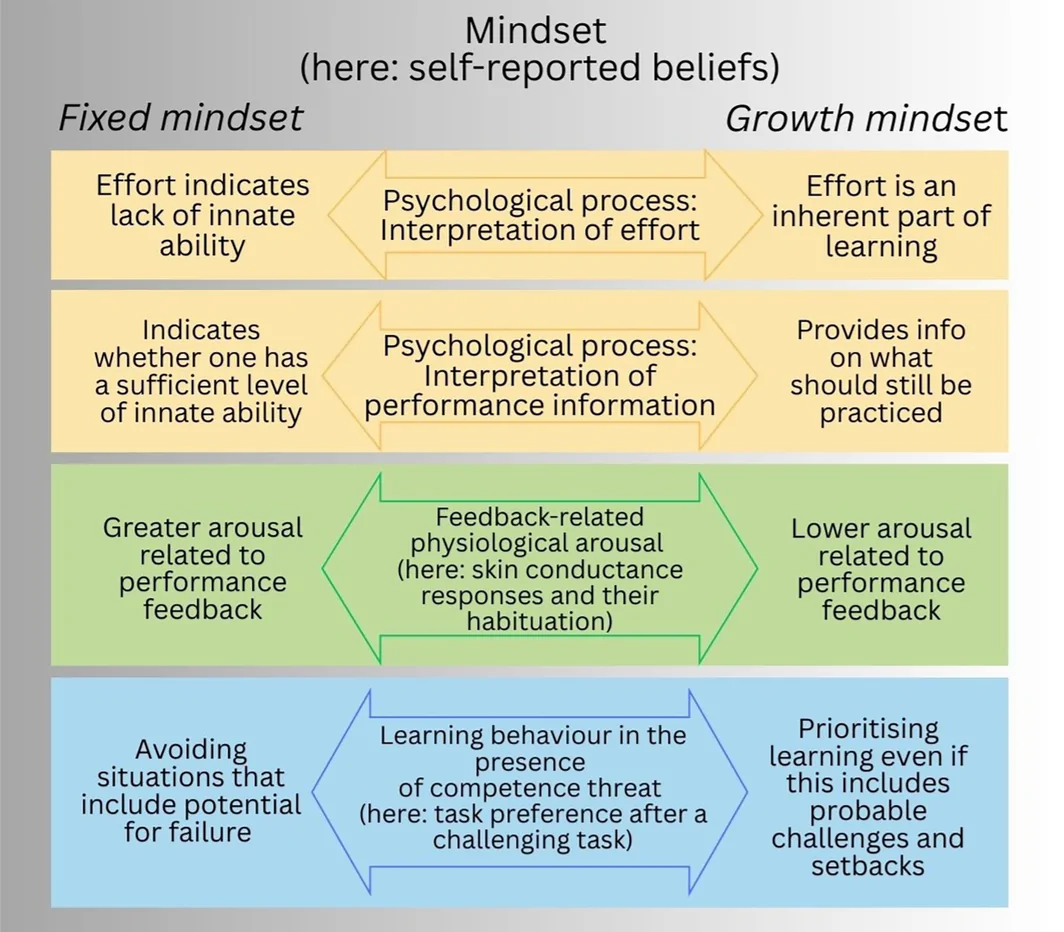
Theoretically assumed differences between a growth and fixed mindset and the operationalization of the focal constructs of the present study.

When examining physiological responses, we focused on (a) the average of participants' feedback‐related SCRs across the task, and (b) habituation of the feedback‐related SCR. The present study was part of the research project Copernicus—Changing Mindsets about Learning: Connecting Psychological, Educational and Neuroscientific Evidence.

The research questions and hypotheses were as follows:

What is the interrelation between students' self‐reported maths ability mindsets, their behaviourally assessed task preference in maths and feedback‐related physiological responses during an arithmetic task?

How do these interrelations develop from one grade year to the next?

We expected (H1a) students with a stronger fixed maths ability mindset to prefer the easy over the challenging maths task (Janssen et al., 2022; see Figure 1). As to responses to feedback, according to the mindset theory, fixed‐minded students view performance feedback as a direct reflection of their fixed ability. Therefore, we expected a stronger fixed mindset to relate to (H1b) heightened arousal when anticipating or receiving performance feedback (here: greater feedback‐related SCRs) and (H1c) a slower habituation of the SCR to feedback (Barry, 2004; Öhman et al., 1974; Puusepp et al., 2023; Walker et al., 2019). However, heightened arousal when faced with info about one's performance (if this arousal reflects the activation of the defensive motivational system) can be expected to lead to avoidance of situations that include potential for failure. Thus, we expected greater feedback‐related SCRs and slower habituation of feedback‐related SCR to also relate to the preference of an easy over a challenging maths task (H1d, H1e, respectively).

When hypothesizing the development of the interrelations between mindset, task preference, and physiological responses, we considered the tentative findings from prior research, which suggest that students' mindsets align more closely with their reactions and behaviours as they mature (Kinlaw & Kurtz‐Costes, 2007; Puusepp et al., 2023). Therefore, we expected (H2) these associations to strengthen over the 1‐year period.

## METHOD

### Participants and procedure

The initial sample comprised 104 participants (51 girls, 48 boys, 5 ‘Other’; *M*_*age*_ at the beginning of the study = 8.94 years) from two Finnish primary schools (*n*_*school A*_ = 56, *n*_*school B*_ = 48) in the Helsinki metropolitan area. The number of participants varied depending on the year and the type of data due to factors such as withdrawal from the study, absence on data collection days, technical issues or noisy physiological data (see Appendix S1).

While one participating school was located in a low socioeconomic status (SES) area, the other was situated in a medium SES area (Vilkama et al., 2014). Parents of 93 participants provided information on the highest maternal education level (basic education or lower *n =* 6; upper secondary education *n* = 23; post‐secondary, university of applied sciences, or Bachelor's degree *n* = 21; Master's degree *n* = 32; Licentiate or Doctoral degree *n* = 11). Students' participation in the study was voluntary, and written parental consent for participation was obtained. Students and parents were informed about the research procedures and their right to withdraw their participation at any moment during the study. The University of Helsinki Ethical Review Board approved the study.

During the autumn terms of their third and fourth grade, the participants' maths ability mindset was assessed with a questionnaire. Then, after working on a challenging maths task, the students indicated which task they preferred to tackle next: another challenging task or an easy task. Participants' physiological data were recorded during individual sessions during the same autumn terms.

### Materials

#### Arithmetic task

EDA was recorded as participants worked on a computerized dichotomous choice arithmetic task consisting of 93 trials (Figure 2). In each trial, the participants judged whether the solution to an arithmetic problem was accurate. Each trial began with an incomplete arithmetic calculation displayed for 3 s. A number then appeared in the place of the missing number for up to 3 s. During these 3 s, participants used their dominant hand to press one of two buttons, indicating whether the number appearing in the calculation was accurate or inaccurate. Following this response, the accurate answer was displayed in bold for 3 s (for the inaccurate trials, the accurate answer replaced the inaccurate one). Participants' incorrect responses were immediately followed by a 100 ms feedback tone. If participants did not respond within 3 s, a time‐out message appeared for 3 s before the next trial began.

**FIGURE 2:**
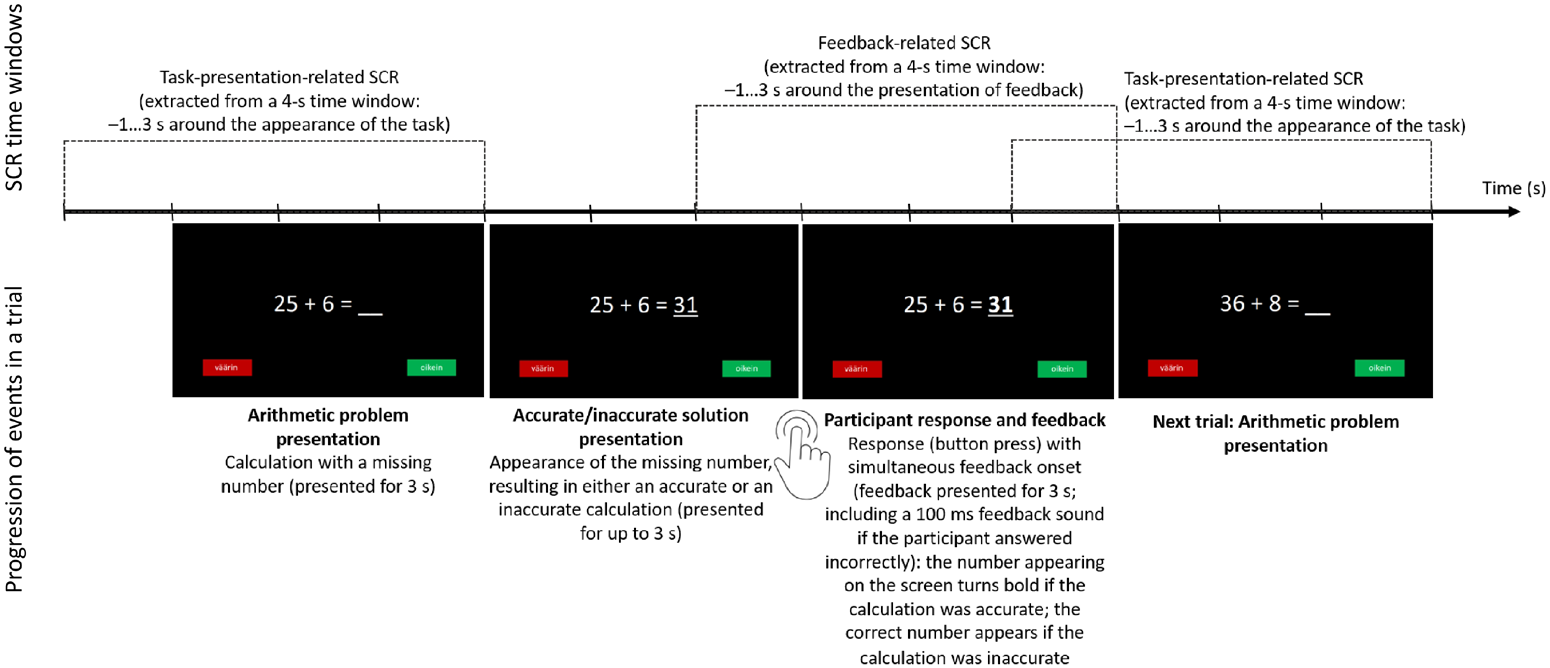
The progression of a single trial in the arithmetic task (from the presentation of the arithmetic problem and its solution to participant response and performance feedback) and the extracted and analysed SCRs. Modified from previously published figures in Puusepp et al. (2023, 2024).

Before the main task, participants completed 10 practice trials. Based on their performance on these practice trials, they received either an easier (6–10 errors on practice trials) or more difficult (0–5 errors on practice trials) version of the main task to ensure appropriate challenge. The 93 trials (48 accurate, 45 inaccurate) were presented in random order across two blocks (46 and 47 trials, respectively) separated by a short refreshment break. Task difficulty was adjusted by grade: third graders solved only addition and subtraction problems, while fourth graders also completed multiplication and division tasks.

#### Electrodermal activity (EDA)

EDA was recorded using LiveAmp (Brain Products GmbH, Germany) at a 250 Hz sampling rate with two Ag–AgCl electrodes. Electrodes were attached to palmar surfaces on the medial phalanges of the second and fourth finger of the non‐dominant hand using adhesive skin tape and TD‐246 paste (.5% saline in a neutral lotion‐style base; Discount Disposables, USA). EDA data were processed using MATLAB R2019a and R2023b software (Mathworks, Natick, MA, USA) with the Ledalab toolbox (Benedek & Kaernbach, 2010). The signal was down sampled to 10 Hz, visually inspected for artefacts, and decomposed into the phasic and tonic components using Continuous Deconvolution Analysis with a two‐step parameter optimization (Benedek & Kaernbach, 2010). The phasic component (SCRs) was then extracted for further analyses.

#### Mindset

Four entity theory statements, adapted to be math‐specific, from the Implicit Theories of Intelligence Scale (Dweck, 2000) were used (e.g., ‘You have a certain amount of maths intelligence, and you cannot really do much to change it.’). Participants indicated their agreement with each statement by marking one of six circles of varying size corresponding to a 6‐point scale ranging from ‘not at all’ to ‘really a lot’. Higher scores indicated a more growth mindset. Reliabilities were good: Cronbach's α was .79 and .86 in Grades 3 and 4, respectively.

#### Task preference

Prior to indicating their task preference, students first worked on a challenging maths task with a 5‐min time limit. This task consisted of three demanding maths exercises that required problem‐solving. These exercises were chosen, and their level evaluated, by two primary school teachers from one of the participating schools. Subsequently, the participants indicated whether they had found the task easy or difficult. After this, they specified whether they next wanted to work on an easy or challenging task (see Figure S1). To control for performance on the preceding exercises in the analyses, *performance on the challenging maths task* was calculated as the number of correct answers in the challenging task (0–3).

### Data processing

We extracted SCRs from time windows of −1…3 s around the stimuli of positive and negative feedback (see Figure 2). In addition, we extracted SCRs from the same time window around the stimulus of task presentation to enable us to examine students' feedback‐related responses relative to their responses to this task‐relevant stimulus. The −1…3 s time window was chosen because the extracted SCRs reflect both anticipatory SCRs related to and SCRs elicited by the specific events (Dawson et al., 2007, 2011).

To quantify SCR, we accounted for both the number of SCRs as well as the typical response for each stimulus in each trial (for each participant): the median SCR amplitude × number of SCRs (Boucsein, 2012). These SCR indicators were used in subsequent analyses.

### Data analysis

As mentioned earlier, when examining physiological responses, we focused on two aspects: (a) overall feedback‐related SCR and (b) habituation of the feedback‐related SCR. First, we focused on the overall feedback‐related SCR across the task. We averaged participants' raw feedback‐related SCRs (separately for positive and negative feedback) and their raw SCRs related to task presentation across the entire task.^1^ This was performed separately for both grade levels. Subsequently, as we were interested in feedback‐related responses relative to responses to task presentation, we calculated SCR difference scores by extracting the mean SCR to task presentation from (a) the mean positive‐feedback SCR and (b) the mean negative‐feedback SCR. These mean positive‐feedback and negative‐feedback SCR∆ for Grades 3 and 4 were subsequently used in the main analyses. To examine how mindset and task preference were linked to these physiological responses to feedback (RQ1) and whether the links changed with time (RQ2), we constructed a linear mixed model (LMM). The model used the mean SCR∆ as the outcome, with the following fixed effects: grade level (Grade 3 vs. Grade 4), feedback type (negative vs. feedback), mindset, task preference (easy vs. challenging), mindset × grade level, task preference × grade level. To control for potential confounding effects of the number of errors on the arithmetic task on the feedback‐related SCR∆, we included the number of errors and its interaction with feedback type as predictors. Furthermore, performance on the challenging maths task preceding the indication of task preference as well as highest maternal education level were also included as control predictors (see top left of Figure 3). Based on preliminary analyses and prior findings (Puusepp et al., 2023), we excluded the interaction effects of mindset × feedback type and task preference × feedback type from the final models. To account for random effects within participants, a random intercept per participant was included in the model.

**FIGURE 3:**
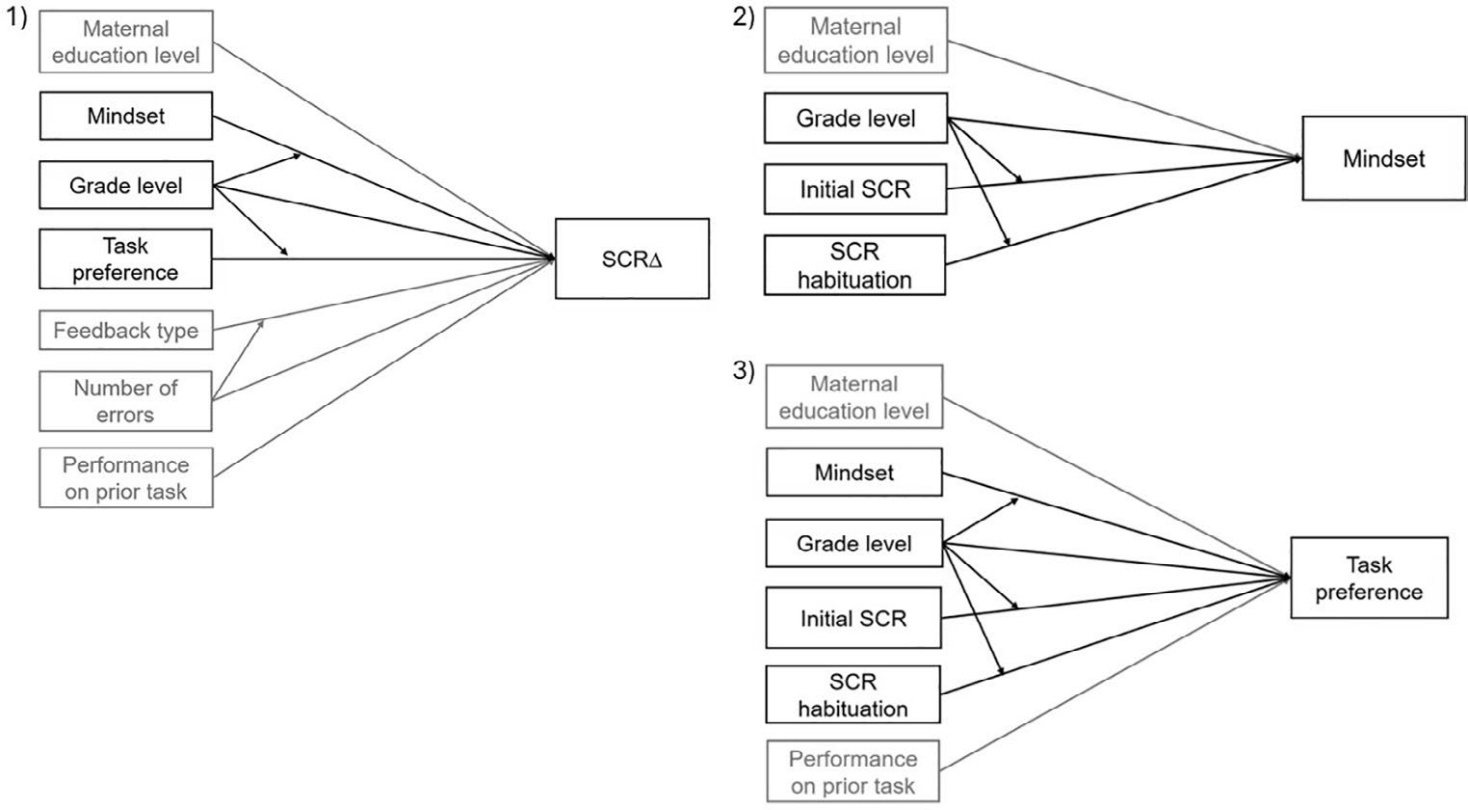
Graphical representation of the three conducted models. The main variables of interest are depicted in black, while the control variables are in grey.

Second, we focused on the habituation of the feedback‐related physiological response.

To examine how mindset and task preference were linked to habituation (RQ1) and whether this association changed with time (RQ2), we first standardized participants' SCRs within individuals separately for Grades 3 and 4. We then used a LMM to determine the habituation of feedback‐related SCR for each participant. In this model, within‐subject standardized feedback‐related SCR was predicted by log‐transformed trial number (base e), with a random intercept and slope for trial number per participant. This LMM was constructed separately for Grades 3 and 4 (Table S1). This allowed us to extract the intercept (initial SCR_1_) and slope (habituation rate) for each participant for both grade levels. Based on visual inspection of SCR habituation (Figure S2), we included trials 1 to 20 in this analysis. Preliminary analyses indicated that performance on the first 20 trials was not related to the SCR_1_ or its habituation (*p* ≥ .232).

Subsequently, we constructed one LMM with mindset as the outcome and one‐mixed effects logistic regression model with task preference as a binary outcome (see top and bottom right of Figure 3). For both models, we included the fixed effects of feedback‐related SCR_1_, its habituation, grade level, grade level × feedback‐related SCR_1_ and grade level × feedback‐related SCR habituation. Highest maternal education level was included as a control predictor. For the task preference model, additional fixed effects included mindset, grade level × mindset, and performance on the prior challenging task preceding task preference indication. To account for random effects within participants, random intercepts per participant were included in these models.

For the mixed effects models, R version 4.5.1 (R Studio Team, 2024) with Rstudio version 2025.5.1.513 (Team Posit, 2025) and the *lme4* (Bates et al., 2015) and *lmerTest* packages (Kuznetsova et al., 2017) were used. When significant effects emerged, estimated marginal means or simple slopes were computed with *emmeans* package (Lenth, 2024). The Bonferroni correction was used to adjust the confidence intervals when post‐hoc tests included multiple tests. Due to the complexity of the models and the small sample size, associations demonstrating marginal significance (i.e. *p* ≤ .10) were also interpreted. To calculate partial eta square effect sizes, the *effectsize* (Ben‐Shachar et al., 2020) package was used and to convert regression coefficients to odds ratios, natural exponential function was used.

## RESULTS

The descriptive statistics are presented in Table 1, while Table 2 shows the bivariate correlations. Mindset scores ranged from fixed to growth, with an overall tendency towards a growth mindset (Table 1). At both grade levels, slightly more than half of the participants opted for the challenging task. Furthermore, the within‐construct correlations over the two measurement points indicate small to moderate stability of mindset, task preference and most physiological responses (Table 2).

**TABLE 1:** Descriptive statistics of study variables.

Variable	*n*	*M*	SD	Min	Max
1. T1 mindset	100	4.15	1.20	1.25	6.00
2. T2 mindset	97	4.35	1.21	1.00	6.00
3. T1 task preference (% preferring challenging task)	100	55	n/a	n/a	n/a
4. T2 task preference (% preferring challenging task)	97	58	n/a	n/a	n/a
5. T1 prior task performance	100	.83	1.04	0	3
6. T2 prior task performance	97	.31	.73	0	3
7. T1 positive‐feedback SCR∆	93	.09	.15	−.18	.53
8. T1 negative‐feedback SCR∆	93	.19	.21	−.13	.76
9. T2 positive‐feedback SCR∆	82	.15	.16	−.15	.65
10. T2 negative‐feedback SCR∆	82	.30	.29	−.09	1.14
11. T1 arithmetic task errors	104	31.99	9.50	10	53
12. T2 arithmetic task errors	99	29.95	8.26	7	49

*Note*: For the feedback‐related SCR∆ presented here, the outliers have already been removed (see Appendix S1 for information on participant exclusion). T1—Grade 3, T2—Grade 4.

**TABLE 2:** Bivariate Spearman correlations. Correlations between grade 3 variables are presented above and Grade 4 variables below the diagonal. Within‐construct correlations are presented in bolded text in the diagonal.

Variable	1	2	3	4	5	6	7	8	9	10	11	12
1. Mindset	.**40** ***	.33***	.18^†^	–.07	–.16	–.11	–.02	–.14	–.09	–.26*	.24*	.30*
2. Task preference	.32**	.**36** ***	.19^†^	–.15	.07	–.03	–.03	> –.01	> –.01	–.04	–.02	.04
3. Prior task performance	.15	.16	.**23** *	–.24*	.01	.09	.06	.02	.07	–.08	.04	.14
4. Arithmetic task errors	–.16	–.24	–.07	.**57** ***	–.02	–.19^†^	–.07	–.02	–.15	–.09	.07	–.23*
5. Positive‐feedback SCR∆	–.02	–.13	.01	–.21^†^	.**50** ***	.73***	.21*	.76***	.64***	.40***	–.24*	–.30**
6. Negative‐feedback SCR∆	–.05	> –.01	–.05	–.28*	.80***	.**49** ***	.42***	.78***	.90***	.39***	–.22*	–.03
7. Task‐presentation SCR	.19	–.03	.05	–.08	.23*	.23*	.**27** *	.75***	.74***	–.05	.04	.06
8. Positive feedback‐related SCR	.11	–.07	.08	–.20^†^	.85***	.85***	.67***	.**40** ***	.92***	.25*	–.16	–.17
9. Negative feedback‐related SCR	.05	.01	< .01	–.28*	.77***	.93***	.54***	.84***	.**43** ***	.24*	–.13	–.02
10. Feedback‐related SCR_1_	–.26*	–.07	.03	.06	.36**	.32*	–.25^†^	.14	.19	.**30** *	–.94***	–.10
11. SCR habituation	.12	–.08	–.07	–.04	–.21	–.21	.21	‐.07	–.12	–.93***	.**20**	.05
12. Highest maternal education level	.42***	.15	.25*	–.11	–.07	< .01	.22^†^	.04	.09	> –.01	–.11	**n/a**

*Note*: Concerning correlations with variables 5 to 9, SCR∆ outliers were excluded prior to calculating the correlations (see also Appendix S1). For task preference, preference for the challenging and easy task was coded as 1 and 0, respectively.

^†^ *p* < .10;

* *p* < .05;

** *p* < .01;

*** *p* < .001.

For feedback‐related SCR∆ (Table 3), the main effects of grade level and feedback type and a significant interaction between feedback type and number of errors was found. A follow‐up analysis indicated that, on average, feedback SCR∆ was larger in Grade 4 than in Grade 3 (Grade 3: EMM = .14, SE = .02; Grade 4: EMM = .24, SE = .02). In addition, on average, the negative‐feedback SCR∆ was greater than the positive‐feedback SCR∆ (EMM = .25, SE = .02 and EMM = .13, SE = .02, respectively). A follow‐up analysis indicated that a greater number of errors was related to a smaller negative‐feedback SCR∆ (*β* = −.05, 95% CI [−.08, −.01]) but was unrelated to positive‐feedback SCR∆ (*β* > −. 01, 95% CI [−.04, .03]). No significant effects involving mindset or task preference emerged (Table 3), which means that at neither grade level were mindset or task preference related to the average feedback‐related SCR∆ during task.

**TABLE 3:** Results of the linear mixed model predicting mean feedback‐related SCR∆.

Fixed predictor	*df*_*Num*_	*df*_*Den*_	*F*	*p*	$\eta_{p}^{2}$
Grade level	1	231.99	31.79	< .001	.13
Feedback type	1	210.93	56.22	< .001	.22
Number of errors (arithmetic task)	1	254.65	3.70	.055	.01
Number of errors × feedback type	1	210.93	6.09	.014	.03
Mindset	1	300.93	.05	.820	<.01
Task preference	1	300.44	1.02	.312	<.01
Performance on prior task	1	300.98	.35	.556	<.01
Mindset × grade level	1	285.03	.67	.435	<.01
Task preference × grade level	1	282.36	.12	.124	<.01
Maternal education level	1	91.29	1.96	.164	.02

*Note*: *n* = 89 participants. Continuous predictors were standardized (repeated measures were standardized within grade level).

The mindset model (Table 4) revealed only a significant main effect of highest maternal education level, with higher level of maternal education being significantly related to a stronger growth mindset (*β* = .35, SE = .11). Based on the task preference model (Table 5), a significant main effect of mindset emerged. More specifically, a stronger growth mindset was linked to preference for the more challenging task across both grade levels. In addition, task preference was linked to the feedback‐related SCR_1_ at the beginning of the task as well as the habituation of this response, although only marginally for the former. More specifically, preference for the more challenging task was marginally linked to a smaller feedback‐related SCR_1_, with this response habituating faster.^2^

**TABLE 4:** Results of the linear mixed model predicting mindset with feedback‐related SCR_1_ and its habituation as predictors.

Fixed predictor	Mindset model				
	*df*_*Num*_	*df*_*Den*_	*F*	*p*	$\eta_{p}^{2}$
Grade level	1	72.87	1.35	.249	.02
Feedback‐related SCR_1_	1	130.89	2.43	.122	.02
SCR habituation	1	130.56	.95	.333	.01
Grade level × feedback‐related SCR_1_	1	114.66	1.44	.233	.01
Grade level × SCR habituation	1	120.15	1.90	.170	.02
Maternal education level	1	92.02	10.41	.002	.10

*Note*: *n* = 88 participants. Continuous predictors were standardized (repeated measures were standardized within grade level).

**TABLE 5:** Results of the mixed effects logistic regression predicting task preference with feedback‐related SCR_1_ and its habituation as predictors.

Fixed predictor	Task preference model				
	*B*/*β*	SE	*z*	*p*	OR
Grade level	.34	.44	.77	.441	1.40
Feedback‐related SCR_1_	−1.43	.86	−1.66	.097	.24
SCR habituation	−1.77	.90	−1.97	.049	.17
Mindset	.73	.34	2.12	.034	2.07
Performance on prior task	.26	.26	1.01	.312	1.30
Grade level × feedback‐related SCR_1_	−1.21	1.55	−.78	.437	.30
Grade level × SCR habituation	−.82	1.55	−.53	.597	.44
Grade level × mindset	−.12	.47	−.25	.805	.89
Maternal education level	−.47	.28	−1.70	.089	.62
Intercept	.26	.30	.87	.385	1.30

*Note*: *n* = 88 participants. Continuous predictors were standardized (repeated measures were standardized within grade level). The model was run using the BOBYQA (bound optimization by quadratic approximation; Powell, 2009) optimizer to avoid non‐convergence.

## DISCUSSION

This study explored the interrelations between primary school students' mindsets, reactions to feedback and task preference in maths from one primary school year to the next. To do this, we combined self‐reported, physiological and behavioural data. Our tentative findings suggest that while students' mindset and learning‐related choices were interconnected, the associations with physiological responses did not emerge or only approached significance in our data. More specifically, we found that a stronger growth mindset was significantly related to preference for the challenging over the easy maths task in Grade 3 and 4 (H1a). In addition, while feedback‐related physiological responses were not related to mindset (H1b, H1c), they were partly and marginally associated with task preference at both grade levels. Only physiological responses at the beginning of the task were related to task preference: students who preferred a challenging over an easy maths task demonstrated a marginally smaller initial feedback‐related physiological response, with this response also habituating faster (H1e). While these results are novel, they require replication and remain highly tentative due to the small present sample size and the limitations of the study design (feedback sound, partial overlap of feedback‐related SCR with other event‐related SCR during task; see Figure 2). Notably, no associations with feedback‐related SCR across the entire task were found (H1b, H1d).

Our finding that students with a stronger growth mindset regarding maths ability preferred the challenging over the easy maths task (H1a) aligns with prior cross‐sectional studies (e.g., Janssen et al., 2022). Students with a stronger growth mindset prioritize learning over maintaining a high‐ability image (Yeager & Dweck, 2020). This link between mindset and behaviour is further suggested to be especially pronounced in situations where students might be required to sacrifice their high‐ability image to learn (Yeager & Dweck, 2020). To our best knowledge, this is the first longitudinal study demonstrating such relations between mindsets and behaviourally assessed learning‐related behaviour across multiple primary school years. However, contrary to our expectation (H2), we observed no increase in the strength of this association over the 1‐year period. In their cross‐sectional study, Kinlaw and Kurtz‐Costes (2007) showed that fourth‐grade students' preference for easy and challenging tasks was linked to their mindsets, while those of second‐grade students and kindergarteners were not. It is possible that by third grade, such associations have already emerged, and a 1‐year period was too short to observe significant changes in the strength of this association in the present small sample. Furthermore, in contrast to Kinlaw and Kurtz‐Costes (2007), our study focused on these links in the context of maths, where the effects of mindset may be more pronounced than in other domains (Janssen & van Atteveldt, 2022). Interestingly, maternal education also positively correlated with student growth mindset. This potentially stems from these mothers' increased use of process‐focused and lower use of person‐oriented feedback (Boncquet et al., 2022).

Our exploration of physiological responses focused on two aspects: the average of participants' feedback‐related SCR across the entire task, and the habituation of this SCR at the beginning of the task. Concerning the former, we examined this average feedback‐related SCR relative to SCR related to the other task‐relevant stimulus—the presentation of the calculation task. Therefore, we examined associations with the SCR difference score (positive/negative feedback‐related SCR minus task‐presentation‐related SCR)—the feedback‐related SCR∆. We found that negative feedback elicited a greater SCR∆ than positive feedback, likely at least partially due to the feedback sound in the case of the former. In addition, we found that the lower the number of errors, the larger the negative‐feedback SCR∆. This indicates that, for students who made less errors during the task, negative feedback was more arousing when compared to students who made more errors. This likely stems from the greater unexpectedness and novelty of negative feedback for higher‐performing students, who encountered such feedback less frequently. It is important to highlight here that these SCRs do not merely reflect the interpretation of feedback, but also arousal triggered by other factors, such as physical properties of the stimuli. Therefore, individual differences in feedback‐related SCR could be reflective of differences in arousal elicited by the physical properties of the feedback (e.g., arousal elicited by the sound accompanying the negative feedback). Interestingly, the feedback‐related SCR∆ was also greater when students were older. Whether this reflects increased subjective importance of performance‐related feedback as students progress in school remains to be confirmed by future studies.

We observed no associations between students' overall feedback‐related SCR∆ and their mindsets or task preference (H1b). Furthermore, the feedback‐related SCR at task onset and its habituation as the task proceeded were not related to students' mindset, but were related to their task preference (H1c). Importantly, the observed associations with task preference were partly marginal and did not differ between grade levels (H2). Specifically, students preferring the challenging over the easy task demonstrated a marginally smaller initial feedback‐related physiological response and a faster habituation of this response in both Grade 3 and 4. While based on theory, speculations concerning the observed associations and the lack of expected associations can be made, it should be emphasized that to draw any actual inferences, the results have to be replicated with a bigger sample as well as a design that minimizes the physical differences between positive and negative feedback and enables better disentangling of feedback‐related physiological responses from other event‐related SCR (e.g., those related to the presentation of other task‐related information).

Based on the mindset theory and earlier research indicating associations between mindsets and attentional neural processing of feedback, we expected to find associations with overall feedback‐related SCR. Specifically, in a prior study among the same sample, we found that in Grade 4, a more fixed mindset was linked to larger feedback‐related brain responses, reflecting fixed‐minded students' greater attention allocation to feedback (Puusepp et al., 2023). This brain response and SCR often co‐occur and are assumed to reflect complementary processes in preparation for action after the perception of a motivationally significant event (Nieuwenhuis et al., 2011). Based on mindset theory, it could be expected that performance‐related feedback holds greater significance for fixed‐minded students, as they should view such feedback as indicative of the level of their innate ability (Dweck et al., 1995). The greater personal significance of the feedback, in turn, could be reflected in greater feedback‐related SCRs. In addition, if greater feedback‐related SCRs reflect greater activation of the defensive motivational system (e.g., fear of failure), we could expect the increased feedback‐related SCR also to be related to greater avoidance of tasks that threaten students' high‐ability image (here, preference for the easy over the challenging task). In this study, however, we only found task preference, but not mindset, to be (partly marginally) linked to the feedback‐related SCRs and only at the beginning of the task (H1d, H1e). It is possible that the connections between mindsets and the feedback‐related brain response found by Puusepp et al. (2023) were more related to differences in attentional processing of the performance feedback (as indicated by the brain responses) and less related to the level of arousal elicited by the performance feedback (as indicated by SCRs). However, even the observed (partly marginal) associations should be interpreted with caution until replicated.

Based on the results concerning feedback‐related SCR at task onset, it is possible that students who found performance feedback to be more arousing and significant (as indicated by SCRs), at least at the beginning of the task, were more concerned about their performance and therefore also opted for an easier task which enabled them to succeed. While this remains a speculation until future replications, the initial SCR has been shown to habituate more slowly when a repeatedly presented stimulus is of greater motivational significance (Barry, 2004; Öhman et al., 1974; see also Bradley, 2009). A greater initial SCR and its slower habituation, albeit to auditory startling stimuli, has also been observed among adults characterized by lower resilience (Walker et al., 2019). We can speculate that these stronger feedback‐related SCRs reflect a greater activation of the defensive motivational system (Bradley, 2009), potentially the fear of failure. However, it is important to note that as SCRs do not enable differentiating between the activation of the defensive system from the appetitive motivational system (Bradley, 2009), this remains purely a speculation. Thus, greater perceived threat of feedback (activation of the defensive system) as well as positive excitement of learning how one performed (activation of the appetitive activation) could both be reflected in a greater initial feedback‐related SCR and its slower habituation. However, it is important to emphasize that our supplementary analyses (Tables S2) indicated that no similar association between task preference and the habituation of the SCR related to task presentation emerged. Thus, while our data do not enable determining which specific motivational system's activation is reflected by greater SCRs, it seems to be performance feedback, specifically, rather than other task‐relevant stimuli, that elicits greater physiological arousal among students preferring an easy over a challenging task.

Interestingly, our findings indicate that self‐reported mindsets and feedback‐related physiological arousal could be uniquely related to behaviourally assessed task preference. Although, the effects of the latter were partly marginal. Furthermore, while task preference was marginally linked to feedback‐related physiological arousal at both grade levels, mindsets were not. This indicates that, at least among this age group, students' feedback‐related physiological responses might be more aligned with their learning‐related behaviour than with their self‐reported mindsets. This may result from the limited self‐reflection skills of students of this age (Borgers et al., 2000), emphasizing the importance of avoiding overreliance on self‐reports and highlighting the potential of including physiological indicators when studying young students. Our results show that students' self‐reported beliefs nevertheless explained variance in their task preference over and above their feedback‐related physiological reactions at both grade levels. While we did not assess students' task preference and their physiological responses in the same situation and the found associations were partly marginal, this result points to the possibility that both students' feedback‐related arousal and their belief in the malleability of their abilities contribute uniquely to their learning‐related decisions. This should be examined in future studies. Namely, students may hold a growth mindset but nonetheless be simultaneously concerned about their performance (e.g., Yu & McLellan, 2020). It is possible that this concern is reflected in students' greater feedback‐related arousal, leading to their preference for easy tasks where success is highly likely.

### Limitations and conclusions

This study has several limitations. First, the arithmetic task included an additional task‐relevant stimulus—the appearance of the missing number in the calculation. We did not examine SCRs related to this stimulus, as they were partly overlapping with those of the feedback stimuli—the participants could respond as soon as the number appeared on the screen. Thus, feedback‐related SCRs in this study might be confounded. Second, SCRs reflect the activation of both the appetitive and the defensive motivational systems (Bradley, 2009). Without self‐reports or physiological indicators that enable disentangling the two (see Blascovich, 2008), we can only speculate which system was activated. Therefore, a heightened feedback‐related SCR could reflect perceived threat of feedback (defensive activation) or positive excitement of learning how one performed (appetitive activation). Importantly, while we focused on SCR as a reflection of the subjective motivational significance of feedback, from a psychophysiological perspective, motivational significance encompasses also factors such as stimulus intensity, unexpectedness and novelty. Consequently, SCRs do not merely reflect the interpretation of feedback, but also arousal triggered by other properties of the stimuli. Third, the small sample size likely limited our power to detect small effects and in combination with multiple analyses the likelihood for Type I errors increased. Thus, our marginal findings require replication with a larger sample. Fourth, negative feedback sound may have confounded the results. However, supplementary analyses (Table S3) found no relationship between feedback frequency and initial SCR or its habituation, supporting the robustness of the results on task preference and SCR in the beginning of the task. Fifth, while the button press immediately preceding feedback might have posed a confound, this is unlikely given that participants used their dominant hand to press the response button, while SCRs were recorded from the resting, non‐dominant hand. Nonetheless, future studies could isolate these effects by introducing a delay between the button press and the feedback. Finally, since physiological responses and task preference were not assessed in the same situation, our conclusions are limited.

Nevertheless, we demonstrated that primary school students' mindsets and learning‐related choices are interconnected, with their feedback‐related physiological reactions being only related to the latter. Furthermore, the initial physiological reaction to feedback and its habituation, not the overall response throughout the task, were related to task preference, and even these effects were partly marginal. While this indicates the need for replication before definitive conclusions can be drawn, it highlights the importance of focusing not only on overall physiological reactions, but also subtle changes in these reactions—like habituation—during educational activities. Such findings could also be relevant for educators, helping to identify specific moments in the learning process where students may need greater support. For example, if future research replicates the present findings with physiological indicators enabling confirmation of the activation of the defensive motivational system, it would highlight the importance of helping students manage performance‐related arousal, particularly as they begin with a task.

## AUTHOR CONTRIBUTIONS

**Ita Puusepp:** Conceptualization; methodology; writing – original draft; visualization; formal analysis; funding acquisition. **Tuisku Tammi:** Methodology; formal analysis; writing – review and editing; conceptualization. **Tanja Linnavalli:** Formal analysis; writing – review and editing; supervision. **Minna Huotilainen:** Conceptualization; writing – review and editing; visualization; supervision. **Sonja Laine:** Writing – review and editing; visualization; conceptualization. **Kirsi Tirri:** Conceptualization; visualization; supervision; project administration; writing – review and editing; resources.

## CONFLICT OF INTEREST STATEMENT

The authors declare no conflicts of interest.

## Supporting information


**Appendix S1.** Participant exclusion from analyses.
**Figure S1.** The challenging maths task, indication of perceived difficulty and task preference (each presented on a separate page of the online survey, translated from Finnish; the maths task presented here is the task utilized in grade 4 data collection).
**Figure S2.** Task‐presentation‐ and feedback‐related SCRs across the 93 trials of the arithmetic task: Means and standard deviations.
**Table S1.** Linear mixed models used for extracting feedback‐related SCR_1_ and the habituation slope.
**Table S2.** Results of the mixed effects logistic regression predicting task preference with task‐presentation‐related SCR_1_ and its habituation as predictors.
**Table S3.** Results of the linear mixed model predicting the number of errors on the first 20 trials of the arithmetic task with feedback‐related SCR_1_ and its habituation as predictors.

## Data Availability

The raw data supporting the conclusions of this article will be made available by the authors, without undue reservation.

## Footnotes

^1^
Trial 1 and 47 SCRs were excluded when calculating mean task‐presentation‐related SCR as these demonstrated extremely deviant SCRs (see Figure S2).

^2^
Additional analyses indicated that no similar associations with task preference emerged when analysing SCR_1_ related to the presentation of the calculation task and the habituation of this task‐presentation‐related SCR (Table S2).
