# Supplementary material for: Associations between maths ability mindset, maths task preference and physiological responses to feedback during an arithmetic task across two primary school years
Source: Br J Educ Psychol. 2026 Aug 7;96(4):1785–801. doi: 10.1111/bjep.70115 (PMC13752640; doi:10.1111/bjep.70115)
Supplement: Supplementary file 1 [file BJEP-96-1785-s001.docx]

**Supplementary materials**

**Appendix A Participant exclusion from analyses**

The criterion for analysis of physiological data was information on at least 50% of EDA throughout the entire arithmetic task for across-task-averaged SCR, which resulted in 96 and 86 students in grade 3 and 4, respectively. In addition, four outliers with across-task-averaged SCR∆ values beyond ±3 SDs were excluded, which resulted in 92 students with data on across-task-averaged SCR∆. Due to additional participants with missing data on highest maternal education level, the final analysis on across-task-averaged SCR∆ included data on 89 participants.

Concerning the analysis on the habituation of the feedback-related SCR, we included participants with at least 50% of data from the first 20 trials in at least one grade level. This resulted in SCR habituation data from 90 and 64 students in grade 3 and 4, respectively. Due to missing data on highest maternal education level, the final LMMs with mindset and task preference as outcomes included data from 88 participants.

**Figure S1**

*The challenging maths task, indication of perceived difficulty and task preference (each presented on a separate page of the online survey, translated from Finnish; the maths task presented here is the task utilised in grade 4 data collection).*

**Figure S2**

*Task-presentation- and feedback-related SCRs across the 93 trials of the arithmetic task: Means and standard deviations*
 *Note.* There was a small pause between the 46^th^ and 47^th^ trial.

**Table S1**

*Linear mixed models used for extracting feedback-related SCR_1_ and the habituation slope*

|  | Grade 3 | | | | | Grade 4 | | | | |
| --- | --- | --- | --- | --- | --- | --- | --- | --- | --- | --- |
| Fixed effects | *B* | *S.E.* | *df* | *t* | *p* | *B* | *S.E.* | *df* | *t* | *p* |
| Intercept | 1.02 | 0.11 | 92.65 | 8.97 | < .001 | 0.87 | 0.13 | 64.73 | 6.62 | < .001 |
| log(trial number) | –0.49 | 0.04 | 93.26 | –10.96 | < .001 | –0.46 | 0.06 | 66.06 | –8.39 | < .001 |

*Note.* Random intercept *SD* = 0.90/0.82, random slope of log(trial number) *SD* = 0.32/0.34, residual *SD* = 0.96/0.95 in grade 3/grade 4, respectively.

**Table S2**

*Results of the mixed effects logistic regression predicting task preference with task-presentation-related SCR_1_ and its habituation as predictors*

|  | Task preference model | | | | |
| --- | --- | --- | --- | --- | --- |
| Fixed predictor | *B/β* | *S.E.* | *z* | *p* | *OR* |
| Grade level | 0.36 | 0.41 | 0.89 | .374 | 1.44 |
| Feedback-related SCR_1_ | 0.60 | 1.51 | 0.40 | .689 | 1.83 |
| SCR habituation | 0.76 | 1.50 | 0.45 | .656 | 2.14 |
| Mindset | 0.52 | 0.30 | 1.75 | .081 | 1.67 |
| Performance on prior task | 0.31 | 0.25 | 1.24 | .216 | 1.37 |
| Grade × feedback-related SCR_1_ | –0.20 | 1.84 | –0.11 | .915 | 0.82 |
| Grade × SCR habituation | –0.58 | 1.83 | –0.32 | .750 | 0.56 |
| Grade × mindset | 0.06 | 0.43 | 0.15 | .883 | 1.07 |
| Highest maternal education level | –0.32 | 0.25 | –1.28 | .201 | 0.73 |
| Intercept | 0.23 | 0.28 | 0.83 | .410 | 1.26 |

*Note.* Continuous predictors were standardized within grade level.

**Table S3**

*Results of the linear mixed model predicting the number of errors on the first 20 trials of the arithmetic task with feedback-related SCR_1_ and its habituation as predictors*

| Fixed predictor | *df_Num_* | *df_Den_* | *F* | *p* |
| --- | --- | --- | --- | --- |
| Grade level | 1 | 92.81 | 2.71 | .103 |
| Feedback-related SCR_1_ | 1 | 145.57 | <0.01 | .965 |
| SCR habituation | 1 | 147.95 | 0.01 | .933 |
| Grade × feedback-related SCR_1_ | 1 | 136.88 | 0.13 | .717 |
| Grade × SCR habituation | 1 | 143.09 | 1.13 | .291 |

*Note.* Continuous predictors were standardized within grade level.
